# A hierarchical signal detection model with unequal variance for binary responses

**DOI:** 10.3758/s13423-024-02504-5

**Published:** 2024-05-28

**Authors:** Martin Lages

**Affiliations:** School of Psychology and Neuroscience, University of Glasgow, 62 Hillhead Street, Glasgow G12 8QQ Glasgow, UK

**Keywords:** Bayesian inference, MCMC sampling, Bivariate normal distribution, Latent parameter estimation

## Abstract

Gaussian signal detection models with equal variance are commonly used in simple yes–no detection and discrimination tasks whereas more flexible models with unequal variance require additional information. Here, a hierarchical Bayesian model with equal variance is extended to an unequal-variance model by exploiting variability of hit and false-alarm rates in a random sample of participants. This hierarchical model is investigated analytically, in simulations and in applications to existing data sets. The results suggest that signal variance and other parameters can be accurately estimated if plausible assumptions are met. It is concluded that the model provides a promising alternative to the ubiquitous equal-variance model for binary data.

## Introduction

Signal detection theory has a long tradition as a psychophysical and cognitive model because it provides a general framework for studying sensitivity and response bias under uncertainty (Fechner, 1860; Tanner & Swets, 1954; Green & Swets, 1966; Falmagne, 1985; Macmillan & Creelman, 2005). In a standard signal detection paradigm, each participant gives binary responses in a series of randomised signal and noise trials. The responses are aggregated and summarised in a confusion matrix that reports the number of hits, misses, false alarms, and correct rejections. The main parameters of interest are typically sensitivity and response bias in a detection or discrimination task. Both parameters can be estimated in a signal detection model where signal and noise distribution overlap on a common decision axis (e.g., Wickens, 2002).

**Fig. 1:**
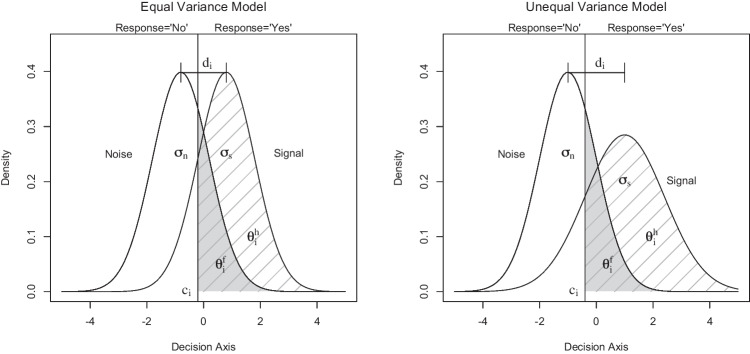
Illustration of signal detection models with equal variance $\sigma ^2_n=\sigma ^2_s$ (*left panel*) and unequal variance $\sigma ^2_s > \sigma ^2_n$ (*right panel*) for a single participant *i*. The distance between the mean of the signal and noise distribution defines sensitivity or discriminability $d_i$. The response bias or criterion $c_i$ separates the two response categories and is defined here as the deviation from the midpoint 0 between the mean of the signal and noise distribution. Hit rate $\theta ^h_i$ and false-alarm rate $\theta ^f_i$ correspond to the *hatched area* under the signal distribution and the *grey area* under the noise distribution, respectively

Gaussian signal detection models are widely used to estimate sensitivity and response bias. They fall into two main categories – models with equal and models with unequal variance (Green & Swets, 1966). Equal-variance models assume that signal and noise distributions have different mean values, yet the same variance. Signal detection models with unequal variance are more flexible because they introduce signal variance as an additional free parameter (Fig. 1) and therefore can accommodate not only symmetric but also asymmetric receiver operating characteristics (ROCs). Asymmetric ROCs have been observed in a number of domains and usually give parameter estimates that are less biased than their equal-variance counterparts (e.g., Ratcliff et al. 1992; Starns and Ratcliff, 2014; Jang et al., 2009; Starns et al., 2014; Spanton and Berry, 2020).

However, estimating the parameters of an unequal-variance model is not always possible. For a single participant, for example, additional information is required because three unknown parameters cannot be estimated from just two observed variables, such as hits and false alarms. Accordingly, researchers often settle for an equal-variance model rather than collect additional data. In experimental paradigms, where each participant performs under multiple conditions, an individual ROC can be approximated from the observed hit and false-alarm rates across conditions. If, for example, rewards, penalties, or proportions of signal and noise trials are systematically manipulated within participants then an individual ROC for unequal variance can be fit to the binary data of each participant.

In other paradigms, researchers have used magnitude estimation (Stevens, 1957), confidence ratings (Dorfman & Alf, 1969), as well as choices and response times (Starns & Ratcliff, 2014; Weidemann & Kahana, 2016) to establish individual ROCs and to fit a signal detection model with unequal variance. Although confidence ratings and continuous response scales are very popular, they are prone to context effects. Moreover, the measurement-theoretic assumptions of ordinal or interval-scaled rating data are debated (Stevens, 1957; MacKay, 1963; Narens, 1996; Luce, 2002; Bürkner & Vuorre, 2019; Kellen et al., 2021).

Nonetheless, researchers are usually less interested in individual performances and more in the overall performance of a sample of participants. Aggregating data across participants, items, and other factors can lead to biased parameter estimates, whereas hierarchical models can exploit these factors (e.g., Gelman et al., 2013). In general, hierarchical models improve statistical inference because partial pooling and shrinkage towards a population mean makes parameter estimates more robust and less susceptible to noise and outliers (Efron & Morris, 1977). For rich data sets, such as confidence ratings from many trials, items and participants, various hierarchical signal detection models with unequal variance have been developed. These models are superior to equal-variance models because they estimate sensitivity, criterion and signal variance at the individual and population level, usually providing better fits and more reliable parameter estimates (e.g., Morey et al. 2008; DeCarlo, 2011; Pratte et al. 2010; Selker et al. 2019; Fleming, 2017; Bürkner and Vuorre, 2019; Paulewicz and Blaut, 2020).^1^

To the best of my knowledge, however, no hierarchical signal detection model with unequal variance has been suggested that can accommodate binary response data from a single condition. Rather than observing hits and false-alarm rates across multiple conditions or multiple response categories, variability of hit and false-alarm rates across participants can be used to estimate signal variance at the population level.

This appears to be at odds with conventional wisdom whereby parameters of an unequal-variance model are not identifiable if only binary responses are observed in a single condition (e.g., Wickens, 2002; p. 50). Although this is true for non-hierarchical signal detection models that estimate individual parameters, a hierarchical Bayesian model can exploit characteristics of sampling distributions to estimate latent parameters at the population level. More specifically, if the independent sample of sensitivity and criteria across participants is sufficiently large and if the midpoint between the mean of signal and noise distributions is used to describe response bias in a single condition then equal signal and noise variance should perturb variability of hit and false-alarm rates to the same extent. If, however, individual signal and noise distributions have unequal variances, that are approximately the same for all participants, then the ratio of the sample variance between (*z*-transformed) false-alarm and hit rates reveals the scaling of the signal distribution relative to the noise distribution. As a consequence, it should be possible to estimate signal variance at the population level from the variability of binary response data.

**Fig. 2:**
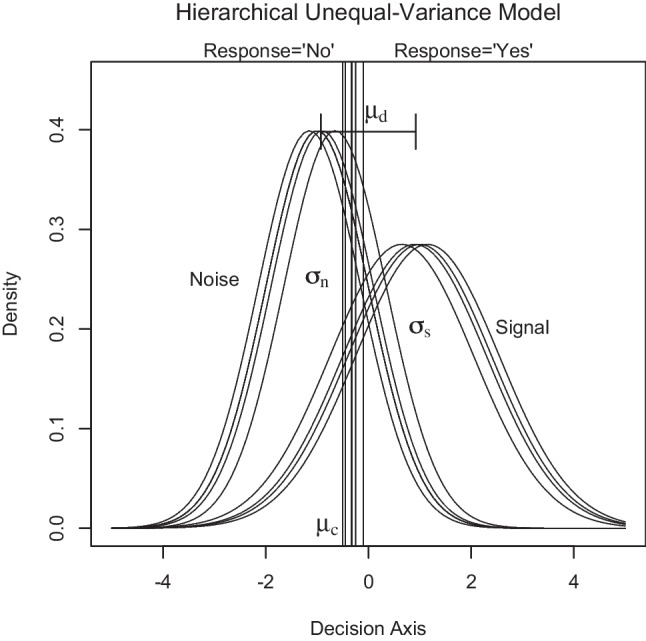
Illustration of a hierarchical signal detection model with unequal variance $\sigma ^2_s > \sigma ^2_n$. Here, sensitivity or discriminability $\mu _d$ and response bias or criterion $\mu _c$ (*vertical thick line*) are the average $d_i$ and $c_i$ of four participants. Note that standard deviation $\sigma _s$ and $\sigma _n$ of the individual signal and noise distributions remain the same for each participant whereas individual sensitivity or discriminability $d_i$ and criteria $c_i$ vary across participants

A Bayesian signal detection model that can accommodate equal as well as unequal variances is more flexible and may produce more accurate parameter estimates compared to an equal-variance model. Aggregating information across rather than within participants is also of practical relevance because sensitivity, response criterion and signal variance can be estimated for separate experimental conditions, providing a more detailed description of performance at the population level. At the same time, this may avoid problems that can arise when aggregating information within participants in order to establish individual ROCs. Besides, it is simpler to instruct and collect data from participants in a binary decision task, and it is easier to estimate and interpret a single response criterion compared to multiple response thresholds.

In the following, it is shown how signal variance together with sensitivity and criterion can be estimated in a hierarchical Bayesian signal detection model that assumes approximately constant signal variance as well as independent normal distributed sensitivity and criterion values in a random sample of participants (Fig. 2). Possible applications of this hierarchical unequal-variance signal detection (hUVSD) model concern binary response data collected from a random sample of participants, subjects or other independent units. Therefore, the hUVSD model may be of interest to psychologists, behavioral scientists, neuroscientists, and biomedical researchers working in different fields.

First, the hierarchical equal-variance signal detection hEVSD model as suggested by Lee (2008) and Lee and Wagenmakers (2013) is described. In the next step, this model is extended to a hUVSD model by introducing signal variance at the population level as an additional scaling parameter.

## Hierarchical equal-variance model

The hierarchical equal-variance signal detection model (hEVSD) is based on binary responses in signal and noise trials from participant *i* in a random sample of $i=1,\ldots , n$ participants (Lee & Wagenmakers, 2013; Chap. 11). Observed number of hits $h_i$ and false alarms $f_i$ are divided by the number of signal $s_i$ and noise $n_i$ trials, respectively, and the resulting rates feature as individual parameter estimates $\theta ^h_i$ and $\theta ^f_i$ of two independent binomial distributions. Next, the rate parameters $\theta ^h_i$ and $\theta ^f_i$ are re-parameterised into sensitivity or discriminability $d_i$ and criterion $c_i$ of a Gaussian signal detection model that is based on standard Gaussian cumulative distributions for signal and noise with equal variance (left panel of Fig. 1). Sensitivity $d_i$ is defined as the distance between signal and noise distribution for each participant *i*. The threshold or criterion at which participant *i* selects a Yes or No response is denoted by $c_i$. Here, criterion $c_i$ is expressed as the deviation from the midpoint 0 between the mean of the noise distribution $-d_i$/2 and the mean of the signal distribution $+d_i$/2.

**Fig. 3:**
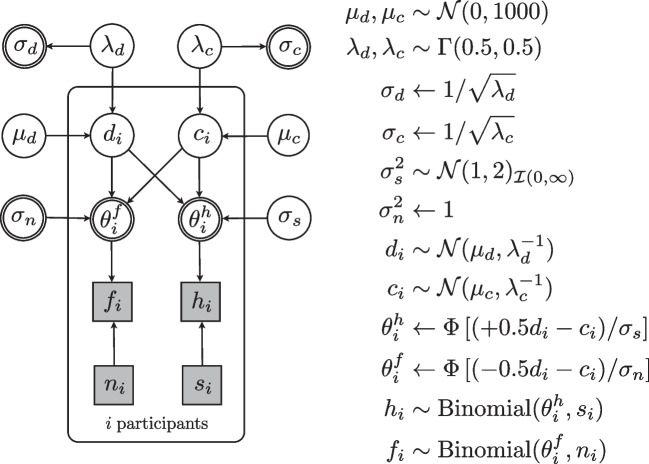
Graphical representation of the hierarchical unequal-variance signal detection (hUVSD) model. The model is a straightforward extension of the hierarchical equal-variance models proposed by Lee (2008) and Lee and Wagenmakers (2013). *Square* and *circular nodes* describe discrete and continuous variables, *gray* and *white nodes* indicate observed and latent variables, respectively. Nodes with *double circles* are continuous variables that are known or can be derived from other variables. *Arrows* indicate parent–child dependencies in the directed acyclic graph. A rectangular plate surrounds the nodes that are here indexed by $i=1 \dots n$ participants

In a linear parameter expansion of the hEVSD model, individual sensitivity $d_i$ and criterion $c_i$ may be defined as

1 $$\begin{aligned} d_i= & {} \mu _d + \xi _d \delta ^d_i \nonumber \\ c_i= & {} \mu _c + \xi _c \delta ^c_i \end{aligned}$$

where $\delta ^d_i$ has a normal distribution with mean 0 and precision $\lambda _d$, the reciprocal of variance $\lambda ^{-1}_d$. The precision $\lambda _d$ of the normal distribution has gamma (0.1, 0.1) as prior distribution.

2 $$\begin{aligned} \delta ^d_i\sim & {} \mathcal {N}( 0, \lambda ^{-1}_d) \nonumber \\ \delta ^c_i\sim & {} \mathcal {N}( 0, \lambda ^{-1}_c) \end{aligned}$$

Similarly, $\delta ^c_i$ is normally distributed with mean 0 and precision $\lambda _c$. The precision $\lambda _c$ of the normal distribution has a gamma (0.1, 0.1) distribution as prior.

In this expanded hEVSD model (Lee & Wagenmakers, 2013) two auxiliary random variables $\xi _d, \xi _c \sim$ beta (1,1) are introduced in order to reduce convergence issues of the equal-variance model with four population parameters. Introducing an independent random variable in a linear expansion can improve convergence because auxiliary random variables can make a model more flexible so that MCMC sampling does not get stuck in local extrema (Liu et al., 1998). However, even though some population parameters may converge better, $\xi _d$ and $\xi _c$ are not sufficiently identified and have no meaning on their own. Alternatively, if the hierarchical equal-variance model is extended to an unequal-variance model, as shown below, then parameters are easier to interpret and converge better.

## Hierarchical unequal-variance model

The hUVSD model assumes that each individual hit rate $\theta ^h_i$ corresponds to an area under a signal distribution with variance $\sigma ^2_s$ and each individual false-alarm rate $\theta ^f_i$ to an area under a noise distribution with variance $\sigma ^2_n$. Similar to the hierarchical equal-variance model, both cumulative probability estimates are expressed by distance $d_i$ between the mean of signal distribution at $+d_i/2$ and the mean of the noise distribution at $-d_i/2$. The midpoint serves as a reference for criterion $c_i$ of each participant *i* with discriminability $d_i$. Centring of parameters helps MCMC sampling and estimation. In contrast to the hEVSD model, however, signal variance $\sigma ^2_s$ may be different from noise variance $\sigma ^2_n$. By introducing standard deviation $\sigma _s$ for the signal distribution together with $\sigma _n=1$ for the noise distribution the hierarchical equal-variance model with four population parameters (Lee & Wagenmakers, 2013) is extended to the hUVSD model with five parameters whereas the expanded hierarchical equal-variance model with six parameters is reduced to five parameters.

Binomial hit and false-alarm rates of each participant *i* are re-parameterised in a Gaussian signal detection model with unequal variance (Fig. 3). The definition of $c_i$ as a deviation from the midpoint between the mean of the noise distribution ($-0.5\, d_i$) and the mean of the signal distribution ($+0.5\, d_i$) simplifies mathematical arguments because each pair of individual hit and false-alarm rates can be expressed by parameters $d_i$ and $c_i$, scaled by $\sigma _s$ and $\sigma _n=1$, respectively.

3 $$\begin{aligned} \theta ^h_i= & {} \mathrm {\Phi } \left[ ( +0.5 \, d_i - c_i )/\sigma _s \right] \nonumber \\ \theta ^f_i= & {} \mathrm {\Phi } \left[ ( -0.5 \, d_i - c_i )/\sigma _n \right] \end{aligned}$$

$\mathrm {\Phi } \left[ . \right]$ denotes the standard Gaussian cumulative distribution function (cdf). The inverse of the Gaussian cdf $\mathrm {\Phi }^{-1} \left[ . \right]$ for each transformed hit and false-alarm rate is written as $z(\theta ^h_i)$ and $z(\theta ^f_i)$, so that (3) becomes

4 $$\begin{aligned} \sigma _s \, z(\theta ^h_i)= & {} +0.5 \, d_i - c_i \nonumber \\ \sigma _n \, z(\theta ^f_i)= & {} -0.5 \, d_i - c_i \end{aligned}$$

Each transformed hit rate $z(\theta ^h_i)$ is scaled by $\sigma _s$ whereas each transformed false-alarm rate $z(\theta ^f_i)$ is multiplied by $\sigma _n=1$. Re-arranging the two equation systems and solving for $d_i$ and $c_i$ gives

5 $$\begin{aligned} d_i= & {} \sigma _s \, z(\theta ^h_i) - \sigma _n \, z(\theta ^f_i) \nonumber \\ c_i= & {} -0.5 \, [ \sigma _s \, z(\theta ^h_i) + \sigma _n \, z(\theta ^f_i) ] \end{aligned}$$

so that sensitivity and criterion are described by a system of $i=1 \dots n$ pairs of linear equations of transformed hit and false-alarm rates where each $z(\theta ^h_i)$ is scaled by $\sigma _s$ and each $z(\theta ^f_i)$ is multiplied by $\sigma _n=1$.

Individual parameters $d_i$, and $c_i$ have population means $\mu _d$ and $\mu _c$ with variances $\sigma ^2_d$ and $\sigma ^2_c$, respectively. Assuming the two distributions are independent normal then the ratio of signal and noise variance $\sigma ^2_s/\sigma ^2_n$ of the hUVSD model may be approximated by the ratio of the sample variances $\text {var}[z(\theta ^f)]/\text {var}[z(\theta ^h)]$ (Proposition 2 in Appendix A).

A number of different measures of response bias have been suggested (e.g., Macmillan & Creelman, 2005). The most sophisticated measure is based on the log-likelihood ratio $\log (\beta )$ of the density of signal and noise distribution. However, there is little evidence that participants adopt such an ‘optimal’ decision strategy (Green & Swets, 1966, pp.91–94; Rahnev, 2021). In addition, this measure is based on a quadratic function that makes it necessary to estimate two thresholds if signal and noise variance differ substantially (Wickens, 2002; p.160). Otherwise, $\log (\beta )$ can be approximated by parameter estimates of the (h)UVSD model (Wickens, 2002, p.75, Eq. 4.11).

### Bivariate normal distributions

In many signal detection studies and hierarchical models, the variance-covariance matrix of the sampling distributions is not specified at all or only implicitly. Here, independence between randomly sampled sensitivity or discriminability $d_i$ and criterion $c_i$ is stated explicitly. For a single task and condition, sensitivity or discriminability $d_i$ and response criterion $c_i$ are modelled as random draws from independent normal distributions with unknown population means and variances. This can be written as

6 $$\begin{aligned} d_i\sim & {} \mathcal {N}( \mu _d, \lambda ^{-1}_d)\nonumber \\ c_i\sim & {} \mathcal {N}( \mu _c, \lambda ^{-1}_c) \end{aligned}$$

where $d_i$ is a draw from a normal distribution with population mean $\mu _d$ and variance $\lambda ^{-1}_d$ ($=\sigma ^2_d$). Similarly, $c_i$ is a draw from a normal distribution with population mean $\mu _c$ and variance $\lambda ^{-1}_c$ ($=\sigma ^2_c$).

This is a plausible assumption because independence of sensory encoding and cognitive processing makes parameter estimates easier to interpret (Green & Swets, 1966) and the signal detection model simpler and more parsimonious. It is an assumption that appears in various hierarchical models (Lee and Wagenmakers, 2013; Selker et al., 2019; Paulewicz and Blaut, 2020) and theoretical considerations (Lynn and Barrett, 2014; Suero et al., 2018).

7 $$\begin{aligned} \left( \begin{array}{ll} d_i \\ c_i \end{array} \right) \!\sim \! \mathcal {N} ({ \pmb \mu }, {\mathbf \Sigma }), \, \!\text {with} \; { \pmb \mu } \!=\! \left( \begin{array}{ll} \mu _d \\ \mu _c \end{array} \right) , \! {\mathbf \Sigma } \!=\! \left( \begin{array}{lll} \sigma ^2_d &{} \rho \, \sigma _d \sigma _c \\ \rho \, \sigma _c \sigma _d &{} \sigma ^2_c \end{array} \! \right) \end{aligned}$$

Accordingly, sensitivity and criterion are described by a bivariate normal distribution with correlation $\rho =0$ (and covariance $\sigma _{d,c}= \rho \, \sigma _d \sigma _c=0$). A significant positive or negative correlation suggests a response strategy, common among participants, whereby sensitivity systematically affects response bias. In contrast, a number of reports indicate that response bias tends to reflect stable and trait-like idiosyncratic differences (Kantner & Lindsay, 2012, 2014) and that correlations between individual discriminability and criterion values tend to be negligible, small or at most moderate, depending on how well other factors such as unequal variance are accommodated (See et al. 1997; Lynn and Barrett, 2014; Rabe, 2018).

If the sampling distribution of the *z*-transformed rate parameters is approximately bivariate normal then the distribution has five parameters. Dropping index *i*, the vector of transformed hit rates $z(\theta ^h)$ is described by sample mean $z(\bar{\theta }^h)$ and sample variance $\text {var}[z(\theta ^h)]$. Similarly, the vector of transformed false-alarm rates $z(\theta ^f)$ has sample mean $z(\bar{\theta }^f)$ and sample variance $\text {var}[z(\theta ^f)]$. The fifth parameter is the sample covariance $\text {cov}[z(\theta ^h), z(\theta ^f)]$. These sampling characteristics can be used to estimate the five population parameters of the hUVSD model (Proposition 1 in Appendix A). The correspondence between the observable characteristics and latent model parameters ensures identifiability of the model as $n \rightarrow \infty$.

8 $$\begin{aligned} \left( \begin{array}{ll} z(\theta ^h_i) \\ z(\theta ^f_i) \end{array}\! \right)&\!\!\sim \!&\! \mathcal {N} ({ \pmb \mu _z}, {\mathbf \Sigma _z}), \, \!\text {with} \; { \pmb \mu _z} \!=\! \left( \begin{array}{ll} (\mu _d/2 \!-\!\mu _c)/\sigma _s \\ -\mu _d/2 \!-\! \mu _c \end{array} \!\right) , \\ {\mathbf \Sigma _z}= & {} \left( \begin{array}{lll} (\sigma ^2_c + \sigma ^2_d/4)/\sigma ^2_s &{} (\sigma ^2_c - \sigma ^2_d/4)/\sigma _s\\ (\sigma ^2_c - \sigma ^2_d/4)/\sigma _s &{} \,\, \,\,\sigma ^2_c + \sigma ^2_d/4 \nonumber \end{array} \right) \end{aligned}$$

In the present approach, parameters are estimated by MCMC sampling because there is no known analytical solution to the estimation problem of the hUVSD model. More specifically, each observed hit $\theta ^h_i$ and false-alarm rate $\theta ^f_i$ is modelled as a binomial distribution, transformed and re-parameterised according to the constraints of the hUVSD model. Crucially, the model assumes that $d_i$ and $c_i$ are drawn from independent normal distributions with unknown means and variances. Related marginal posterior distributions are described in Appendix A.

### Prior distributions

The normal distributed priors for the population means $\mu _d$ and $\mu _c$ are centred on 0 with large variance, such as $\mathcal {N}(0, 1000)$. Both of these ‘weakly-informative’ priors have negligible effects on parameter estimation.

Following the recommendations for estimating unknown variances in hierarchical models (Gelman, 2006), a conjugate prior for unknown precision of normal-distributed sensitivity and criterion is the gamma distribution, written as gamma($\alpha , \beta$), with shape parameter $\alpha ={\nu }/2$ and inverse scale or rate parameter $\beta =\nu /{2}$. If degrees of freedom for the prior are set to $\nu =1$ then gamma(0.5, 0.5) which is equivalent to a chi-square distribution $\chi ^2_1(1)$ with scale $s^2_{\alpha }=\alpha /\beta =1$ and degrees of freedom $\nu _\alpha =2\alpha =1$. Accordingly, both unknown precision parameters, $\lambda _d$ and $\lambda _c$, have gamma (0.5, 0.5) as a prior.

A normal distribution $\mathcal {N}(1,2)_{I(0,\infty )}$ is suggested as a prior for signal variance $\sigma ^2_s$. The prior is truncated at zero because signal variance cannot be negative. Introducing this ‘informative prior’ may appear contentious. However, this prior distribution favours an equal- over an unequal-variance model because the distribution is centred on 1.0, leading to more conservative estimation of unequal variance. Any departure of $\hat{\sigma }_s$ from 1 is the result of empirical evidence in the data overwhelming the prior. The strength of this evidence depends on the sample variance of hit and false-alarm rates, trial number and sample size. If the sample is large enough then the prior distribution may be changed from an ‘informative’ normal prior to a ‘weakly informative’ gamma or ‘uninformative’ uniform prior with negligible effect on parameter estimation.

## Simulations

The hUVSD model assumes that the five observable parameters of the bivariate distribution of hit and false-alarm rates (two means, two variances, and their covariance) can be re-parameterised in terms of the five population parameters of the hUVSD model. Unlike previous hierarchical models that estimate signal variance at the individual level from variability within participants across response categories or across experimental conditions, the hUVSD model estimates signal variance at the population level from the variability between participants.

In order to benchmark the performance of the hUVSD model a series of simulation studies were conducted in R 3.6.3 (R Core Team, 2020) using different population parameter values to generate data for a sample of synthetic participants. The hUVSD model was implemented in JAGS (Plummer et al., 2006; a clone of BUGS; Lunn et al., 2000) and sampled from 10,000 iterations in four chains with random initialisations of $\lambda _c$ and $\lambda _d$ using the package rjags and runjags in R. The first 4000 iterations (burn-ins) of each chain were removed.

### Parameter recovery

First, parameter recovery for arbitrarily chosen population parameters was investigated. The model converged for each recovered parameter with $\hat{R}<1.01$ (Gelman & Rubin, 1992). Recovery of parameter values $\mu _d=2.0$, $\mu _c=-0.5$, $\sigma _s=1.5$, ($\sigma _n=1.0$), $\sigma _d=0.6$ and $\sigma _c=0.4$ is reported for a sample of $n=500$ synthetic participants with binary responses in $k=400$ trials (200 signal and 200 noise trials). According to the hUVSD model, these population parameters define independent normal distributions for sensitivity or discriminability and for response criterion from which random samples are drawn. Pairs of random values from this sample were then transformed into corresponding hit and false-alarm rates using binomial approximations of Gaussian cumulative distribution functions with unequal variance.

**Fig. 4:**
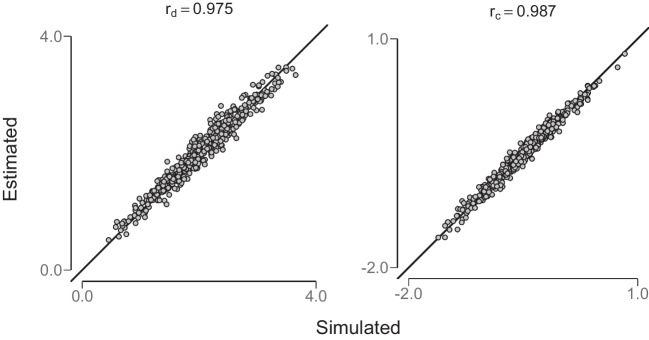
Parameter recovery of the hierarchical unequal-variance signal-detection hUVSD model. The *left panel* shows sensitivity or discriminability $\hat{d}_i$ and the right panel criterion $\hat{c}_i$. These parameters were estimated from simulated data generated from (arbitrary) population parameter values of $\mu _d=2.0$, $\mu _c=-0.5$, $\sigma _d=0.6$, $\sigma _c=0.4$, and $\sigma _s=1.5$ randomly sampled from *n*=500 synthetic participants in *k*=400 trials (200 signal and 200 noise)

In the next step, the hUVSD model was fit to the simulated hit and false-alarm rates of synthetic participants. In Fig. 4, mean estimated posterior values of individual discriminability $\hat{d}_i$ (left panel) and criterion $\hat{c}_i$ (right panel) are compared to the simulated values. For a large sample of *n*=500 synthetic participants, values remain close to the main diagonal. Pearson correlations between estimated and simulated parameter values of $\text {r}_d$ = 0.975 and $\text {r}_c$ = 0.987 with estimated and predicted parameters centred on the main diagonal suggest very good parameter recovery.

Posterior means, estimated from simulated data for criterion values $\mu _c$ ranging from -0.75 to +0.75, are listed in Table 1, for sensitivity or discriminability values $\mu _d$ ranging from 0.5 to 2.5 in Table 2 and for signal variances $\sigma _s$ ranging from 0.5 to 2.5 in Table 3. The simulations illustrate that population parameters can be reliably recovered for a wide range of values without systematically affecting each other.

### Parameter bias

**Fig. 5:**
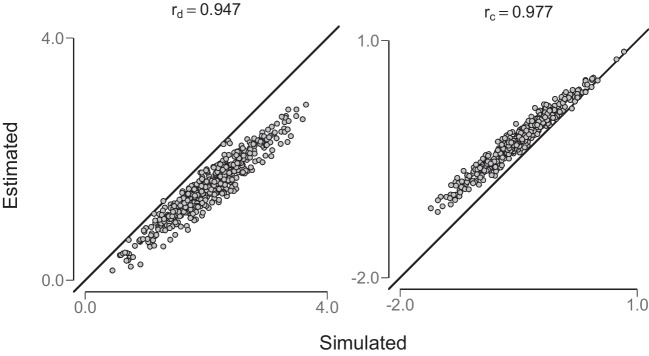
The hierarchical equal-variance signal-detection (hEVSD) model is fit to the same data as in Fig. 4. Parameter estimates of the hEVSD model are systematically biased when fit to data generated by the unequal-variance model

If the hEVSD model rather than the hUVSD model is fit to simulated binary data generated by the unequal variance model with $\sigma ^2_s > \sigma ^2_n$ then estimates are systematically biased, underestimating sensitivity or discriminability and overestimating criterion as shown in the left and right panel of Fig. 5. In contrast, if simulated hit and false-alarm rates are derived from population parameters with equal variance $\sigma ^2_s=\sigma ^2_n=1$ and are fit to the hUVSD model then estimates closely approximate the parameter values, showing no systematic bias.

This illustrates the key advantage of the hUVSD model over the hEVSD model. If the hUVSD model is fit to binary data generated by an equal-variance model then parameter estimates are unbiased and signal variance approximates 1. If, however, the hEVSD model is fit to binary data from an unequal-variance model then sensitivity or discriminability and criterion parameters systematically deviate from the true values (Verde et al., 2006). Corresponding parameter shifts can be expected when the hUVSD and hEVSD model are applied to empirical rather than simulated data. However, for empirical data the true generating model remains unknown.

**Fig. 6:**
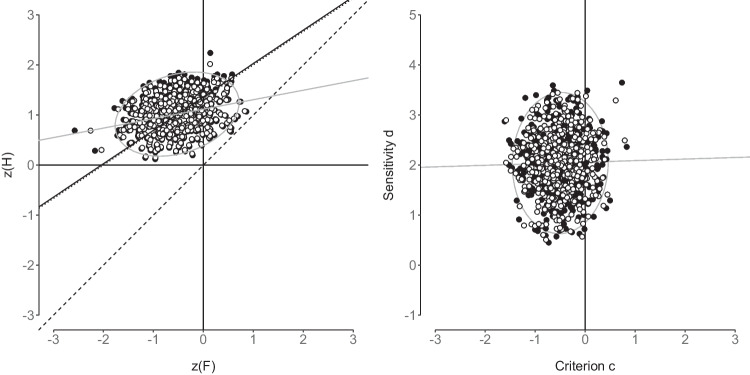
The *graph on the left* shows the receiver operating characteristic (*z*-ROC) for transformed hit and false-alarm rates. *Filled circles* denote simulated data points of *n*=500 synthetic participants and superimposed *open circles *denote the corresponding data points estimated by the hUVSD model. The *solid* and *dotted lines* represent the estimated and true operating characteristic (iso-sensitivity), respectively. The *graph on the right* shows the corresponding estimated and simulated data points for criterion and discriminability. *Gray ellipses* fit 95% of the normally distributed data and *gray lines* represent least-squares regression lines

In Fig. 6 the left panel shows transformed hit and false-alarm rates for *n*=500 synthetic participants from *k*=400 trials (200 signal and 200 noise trials). Each data point denotes a synthetic participant of the hUVSD model. The scatterplot of superimposed estimated (open) compared to simulated (filled) data points illustrates shrinkage towards the mean of the bivariate sampling distribution of (transformed) hit and false-alarm rates. The gray ellipse describes a 95% fit of the bivariate normal distribution and the gray line denotes the least-squares regression. The solid black line corresponds to the estimated and the dotted line to the true *z*-ROC. The operating characteristics were established by inserting model estimates (solid line) and true population parameter values (dotted line) into equation

9 $$\begin{aligned} z(\bar{\theta }^h) = \frac{1}{\sigma _s} z(\bar{\theta }^f) + \frac{\mu _d}{\sigma _s} \end{aligned}$$

where $z(\bar{\theta }^h)$ and $z(\bar{\theta }^f)$ are the sample means of simulated (transformed) hit and false-alarm rates, $1/\sigma _s$ defines the slope, and $\mu _d / \sigma _s$ the intercept of this linear function. Data points that fall onto the iso-sensitivity line describe synthetic participants who have the same sensitivity or discriminability as the population mean but varying criterion values. Figure 6 shows that the linear combination of individual transformed hit and false-alarm rates in the left panel approximate independent normal distributions of sensitivity and criterion estimates in the right panel. In this simulation the sample covariance between $z(\theta ^h)$ and $z(\theta ^f)$= 0.048 which is close to the value that can be derived from the population parameters $(0.4^2 - 0.6^2/4)/1.5$=0.047 (Proposition 1 in Appendix A). The sample covariance between *d* and *c* $\text {cov}[d,c]=-0.0045$ is close to the expected value of 0.

### Parameter robustness

In order to create more realistic data sets, signal variance was varied across participants in the first six simulations. A normal rather than a gamma distribution was used to generate varying individual signal variances $\sigma ^2_{s,i}$. This simplifies introducing a correlation between individual sensitivity $d_i$ and signal standard deviation $\sigma _{s,i}$. In six simulations, both participant and trial number were halved in each subsequent simulation, starting with sample size *n*=512 and trial number *k*=512 (256 signal and 256 noise trials) in the first simulation and with sample size *n*=16 and trial number *k*=16 in the sixth simulation (Tables 4, 5, 6 and 7). As the number of participants and trial numbers was reduced in each simulation, signal variance varied between participants according to sample size *n*. For each sample size, the variability of the signal standard deviation $\sigma _{s,i}$ was matched to the standard error of the sample variance ($\sigma _{s,i} \sim \mathcal {N}(\sigma _s, 2\sigma ^4_s/n)$ with $\sigma _s=1.5$). Otherwise variability of $\sigma _{s,i}$ between participants would exceed the standard error of the sample variance, resulting in extreme hit and false-alarm rates that affect convergence of the model. For each simulated data set, the generated number of hit and false alarms for each participant were fit to the hUVSD and hEVSD model without introducing any ‘edge-correction’ of extreme values.

Although credibility intervals of parameter estimates increase considerably for smaller sample sizes and lower trial numbers the mean posterior estimates suggest that parameter recovery remains robust for a correlation of 0.5 between sensitivity $d_i$ and variable signal standard deviation $\sigma _{s,i}$ (Table  4). A similar result emerged for a correlation of 0.5 between criterion $c_i$ and variable signal standard deviation $\sigma _{s,i}$ (Table 6).

If the hEVSD model is fit to simulated data from the hUVSD model (with the same seed, correlation and sample variance) then the main parameter estimates of sensitivity and criterion are systematically biased, with true parameter values frequently outside the estimated 95% credibility intervals (Table 5).

**Fig. 7:**
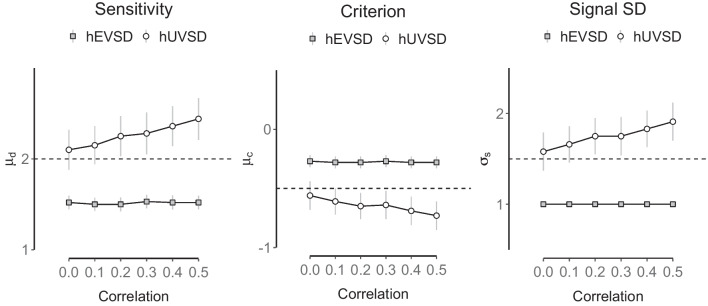
Parameter estimates of sensitivity (*left panel*), criterion (*middle panel*) and signal standard deviation (*right panel*) of the hUVSD model (*white circles*) and hEVSD model (*gray squares*) for simulated data from *n*=64 participants and *k*=64 trials (32 signal and 32 noise trials). MCMC estimates are shown for six correlations between sensitivity and criterion ranging from 0 to 0.5 in steps of 0.1. *Error bars* denote $\pm 1$ *SD* from the posterior mean whereas *dashed horizontal lines* indicate a ‘true’ parameter value of $\mu _d=2.0$, $\mu _c=-0.5$ and $\sigma _s=1.5$, respectively

A violation of independence was also investigated by introducing a correlation between sensitivity or discriminability $d_i$ and criterion $c_i$ in six simulations with a medium number of *n*=64 participants and *k*=64 trials (32 signal and 32 noise trials). The three main parameter estimates of the hUVSD model (white circles) show increasing bias away from the true values (dashed lines in Fig. 7) when the correlation is increased from 0 to 0.5 in steps of 0.1. Sensitivity values and signal variance increase whereas criterion values decrease. Estimates of the hEVSD model (gray squares) are clearly biased at 0 correlation, but are not affected by increasing correlations between $d_i$ and $c_i$.

Interestingly, bias of hUVSD parameter estimates was reduced for smaller sample sizes and reduced trial numbers, most likely because more shrinkage towards the mean lowers the correlation between sensitivity and criterion estimates (Table 7). The hEVSD model produced more stable parameter estimates and was not affected by the correlation between sensitivity and criterion values. However, sensitivity was systematically underestimated and criterion systematically overestimated when signal variance is fixed at 1 (Fig. 7). The simulations suggest that stronger correlations between sensitivity and criterion values introduce bias in the hUVSD model that eventually exceeds the constant bias in the hEVSD model.

These simulations on robustness are limited because all three population parameters (sensitivity, criterion and signal SD) may covary in a hierarchical signal detection model with unequal variance. The true characteristics of the population distributions are unknown and estimates typically depend on the specific model, stimuli, task, conditions and sample of participants. It may be possible to establish these characteristics for rich data sets based on confidence ratings but so far very few empirical studies have investigated and reported estimated variance-covariance matrices.

**Fig. 8:**
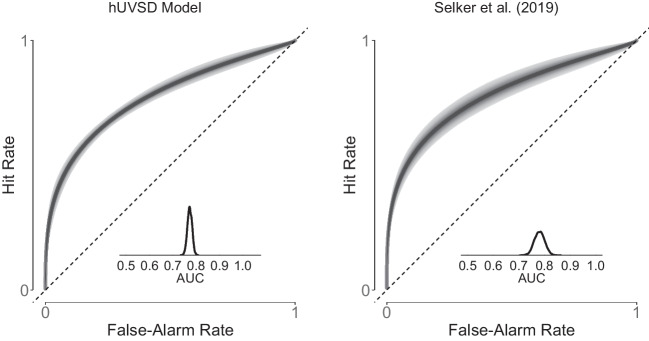
Receiver operating characteristic (ROC) with 95% credible interval in grey and area under the curve (AUC) density distribution using the data from Pratte and Rouder (2011). *Left panel* shows results for the hUVSD model based on dichotomised confidence ratings and the *right panel* results for the hierarchical threshold model (Selker et al., 2019) based on original confidence ratings

## Applications

In the following the hUVSD and hEVSD model is applied to existing data sets. These data sets were selected because they are convenient to use and cover three domains with different tasks, trial numbers and sample sizes.

In the first application parameter estimates of more sophisticated hierarchical models with unequal variance (Pratte et al., 2010; Selker et al., 2019) based on confidence ratings served as a benchmark for the hUVSD model that relies on dichotomised confidence ratings only. In more challenging applications, observed hit and false-alarm rates were estimated from binary data and confidence ratings based on experiments on reasoning tasks with a medium sample size and a small number of trials per participant (Heit & Rotello, 2005).

In the third application, response data came from an auditory discrimination task with five conditions and a small sample size but many trials per participant (Tanner et al., 1967).

### Word recognition

Pratte et al. (2010) collected confidence ratings from *n*=97 participants in a word recognition task. In a study phase, each participant encountered 240 words. In each trial a word was shown for 1850 ms followed by a 250-ms inter-trial interval. The words were randomly selected from a total sample of 480 words. In the subsequent memory test participants had to indicate how confident they were that a word was part of the previously seen study list on a six-point Likert scale using the ratings ’sure new’, ’believe new’, ’guess new’, ’guess studied’, ’believe studied’, and ’sure studied’ for each of the 480 words. In this experiment, 240 words from the study list were used as targets in signal trials whereas 240 words that were not from the study list were used as foils in noise trials. In order to apply the hUVSD model the confidence ratings, ranging between 1 and 6, were dichotomised into a *Yes* response if ratings were $\le 3$ and into a *No* response, otherwise. The dichotomised responses were aggregated across signal and noise trials in order to establish hit and false-alarm rates for each participant.

The hUVSD model was applied to the data set prm09 (R-package hbmem) using JAGS (R-package rjags). Parameters were sampled for 10,000 iterations in 4 chains with random initialisations for $\lambda _d$ and $\lambda _c$. The first 1000 iterations (burn-ins) of each chain were removed. Each parameter of the model converged with $\hat{R}<1.01$. The model fit passed various convergence diagnostics. Changing the prior for signal variance to gamma(0.5, 0.5) or to a uniform distribution dunif (0,5) did not affect mean parameter estimates. Bivariate distributions of estimated population parameters in Fig. 12 show a positive correlation between $\mu _d$ and $\sigma _s$ and a negative correlation between $\sigma _s$ and $\mu _c$ as well as between $\mu _d$ and $\mu _c$.

The receiver-operating characteristic (ROC) and area under the curve (AUC) of the hierarchical threshold model (Selker et al., 2019) are based on the confidence ratings (right panel in Fig. 8) whereas ROC and AUC of the hUVSD model are derived from aggregated hit and false alarms using the dichotomised confidence ratings (left panel in Fig. 8). At the population level, the discriminability estimate of the hUVSD model was $\hat{\mu }_d$=1.31, with 95% credible interval CrI [1.17,1.48] and the estimate of the *SD* of signal distribution was $\hat{\sigma }_s$=1.45, CrI [1.20,1.75] (Table 8). Despite using dichotomised rating data, the estimates closely match the estimates $\hat{\mu }_d=1.38$ and $\hat{\sigma }_s=1.36$ of the best-fitting full hierarchical unequal-variance model with item-, lag- and participant-specific random effects (Model 1) as reported by Pratte et al. (2010). Selker et al. (2019) reported slightly higher values for $\hat{\mu }^{(d)}$=1.41 and $\hat{\sigma }^{(s)}$=1.54 when applying their parsimonious hierarchical threshold model to the original confidence data.

**Fig. 9:**
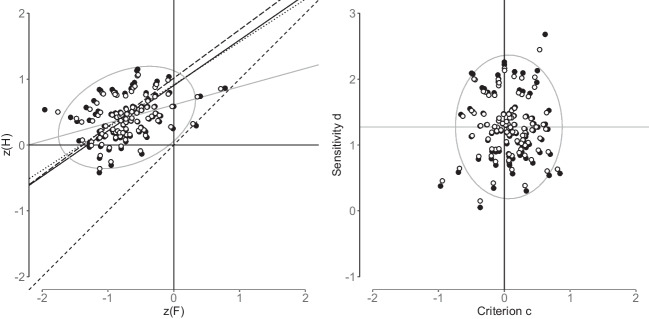
The *graph on the left* shows the receiver operating characteristic (*z*-ROC) for dichotomised and transformed response rates from Pratte et al. (2010). *Black circles* denote observed data of individual participants ($z(\theta ^f_i)$,$z(\theta ^h_i)$) and superimposed *white circles* denote values estimated by the hUVSD model. The *solid*, *dotted* and *dashed line* represent iso-sensitivity (discriminability) of the hUVSD model, the hierarchical UVSD Model 1 (Pratte et al., 2010), and the hierarchical UVSD threshold model (Selker et al., 2019), respectively. The *graph on the right* plots criterion values $c_i$ against discriminability values $d_i$. The *gray lines* represent least-squares regressions and the *gray ellipses* surround 95% of the bivariate normal distribution

In comparison to the hierarchical threshold model (Selker et al., 2019) the ROC (and AUC) of the hUVSD model has smaller credible intervals (Fig. 8). The hUVSD model underestimates variability of the ROC because responses are aggregated across response categories, items and lags (Pratte & Rouder, 2011).

The hUVSD model was also compared to the hEVSD model with equal variance. As illustrated in the simulations (Fig. 5) estimated discriminability $\hat{\mu }_d$ is increased while estimated criterion $\hat{\mu }_c$ is reduced by half the amount for the hUVSD model compared to the hEVSD model (Table 8). The deviance information criterion (dic; Spiegelhalter et al., 2002; Plummer, 2008) was employed to compare model performance. The small difference between dic=1469 for the hUVSD model and dic=1470 for the hEVSD model suggests equivalent model performance. The model fit for the hUVSD and hEVSD model was replicated using a Metropolis–Hamiltonian sampler STAN (R-package rstan; Stan Development Team, 2019). This gave almost identical parameter estimates (Table 8) with a negligible difference between the widely applicable information criteria (waic) as performance measure (Watanabe, 2010).

In the *z*-ROC graph of Fig. 9 individual responses are dichotomised, aggregated over trials in hit and false-alarm rates and then transformed into *z*-values. Each data point denotes a different participant. The scatterplot of observed (filled) with estimated (open) data points superimposed illustrates shrinkage in the hUVSD and hEVSD model. The solid, dotted and dashed line describe iso-sensitivity (discriminability) as predicted by the hUVSD model, hierarchical UVSD Model 1 (Pratte et al., 2010) and hierarchical UVSD threshold model (Selker et al., 2019), respectively. Each of the linear functions was established by inserting population parameter estimates of the corresponding hierarchical model into (9).

In the present example (Fig. 9), the solid line of the hUVSD model has a slope of $1/1.45=0.69$, crosses the *x*-axis at $x_0=-1.31$ and intersects the *y*-axis at $y_0=0.90$ with a slope of $1/1.45=0.69$. The hUVSD function is reasonably close to the hierarchical unequal variance model as reported by Pratte et al. (2010) and by Selker et al. (2019). In contrast to the other models, the hUVSD model uses only dichotomised confidence ratings and is based on a single population estimate of signal variance.

**Fig. 10:**
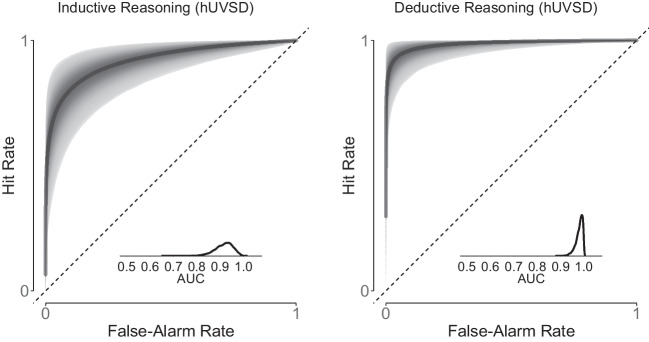
Receiver operating characteristic (ROC) with 95% credible interval in *grey* and density of area under the curve (AUC) for data from Heit and Rotello (2005). The hUVSD model was applied to binary responses of the inductive (*left panel*) and deductive reasoning task (*right panel*)

Finally, the posterior mean of parameter estimate $\hat{\mu }_d$=1.31, $\hat{\sigma }_d=0.49$, $\hat{\mu }_c$=0.05, $\hat{\sigma }_c$=0.36 and $\hat{\sigma }_s$=1.45 were used to create normal distributions for discriminability and for criterion from which pairs of random probabilities were independently drawn to simulate the performance of *n*=97 synthetic participants. Each pair of random values was approximated by a corresponding hit and false-alarm rate. As in the previous simulations the hUVSD model was then fit to the simulated hit and false-alarm rates, drawing samples of posterior values at the individual and population level. In Fig. 13, mean estimated posterior values of individual discriminability $d_i$ (left panel) and criterion $c_i$ (right panel) were compared to the generated posterior values. Pearson correlations of $\text {r}_d$ = 0.977 and $\text {r}_c$ = 0.987 between estimated and generated parameter values suggest very good parameter recovery for the simulated data with $k=240$ trials and $n=97$ participants, matching the bivariate distribution of the empirical data.

In Fig. 14 predicted hit and false-alarm rates, generated from the population parameter values above, are plotted against observed hit and false-alarm rates as reported by Pratte et al. (2010). If predicted and observed hit rates (left panel) and false-alarm rates (right panel) are independently rank-ordered then Pearson correlations reach values of $\text {r}_h$=.994 and $\text {r}_f$=.984, respectively.

### Inductive and deductive reasoning

The data sets are from an empirical study by Heit and Rotello (2005) who investigated a conjecture by Rips (2001). They proposed that inductive and deductive reasoning may be described in a signal detection framework where the strength of arguments is expressed on a single decision axis. According to this approach, deductive reasoning is viewed as a more stringent form of inductive reasoning. More specifically, if discriminability and criterion are independent of each other, then deductive reasoning should shift the decision criterion of inductive reasoning without affecting discriminability. This result would have implications for our understanding of reasoning (Oaksford & Chater, 2007).

Heit and Rotello (2005) collected binary decisions followed by confidence ratings (1 to 7) for 4 strong and 4 weak arguments. Despite a reasonable sample size the low number of signal and noise trials gave rise to very coarse hit and false-alarm rates, making a hUVSD model fit challenging. They employed a between-subjects design so that *n*=40 participants judged whether the conclusion of each argument was *strong* or *not strong* in the inductive reasoning task while another sample of *n*=40 participants judged whether the conclusion of each argument was *valid* or *not valid* in the deductive reasoning task. Each data set was fit separately by the hUVSD and hEVSD model for binary data. The results are summarised in Table 9.

As before, the hUVSD and hEVSD model was implemented in JAGS (R-package rjags). MCMC sampling was conducted with 10,000 iterations in each of four chains and random initialisation of $\lambda _d$ and $\lambda _c$. The first 1000 iterations (burn-in) of each chain were removed. Although all parameters of the model converged with $\hat{R}<1.01$ sampling was less efficient and precise as in the previous application. The bivariate posterior distributions of population parameter estimates in Figs. 15 and 16 in the Appendix show a positive correlation between $\mu _d$ and $\sigma _s$ and negative correlations between $\mu _d$ and $\mu _c$ as well as $\sigma _s$ and $\mu _c$. Although the bivariate posterior distributions for inductive reasoning in Fig. 15 and deductive reasoning in Fig. 16 look similar their parameter values are quite different.

ROC and AUC of the hUVSD model are based on hits and false alarms for inductive and deductive reasoning (Fig. 10). For inductive reasoning, mean posterior discriminability of the hUVSD model is $\hat{\mu }_d$=2.64 with 95% credible interval CrI[1.46,3.98]. For deductive reasoning, discriminability is estimated as $\hat{\mu }_d$=4.19 with credible interval CrI[2.76,6.12]. For inductive as well as deductive reasoning, the standard deviation of the signal distribution is $\hat{\sigma }_s$=1.57 with CrI[0.94,2.18] and $\hat{\sigma }_s$=1.63 with CrI[1.05,2.21], respectively. Signal variance is clearly larger than 1.0, close to 1.6 in the inductive as well as deductive reasoning task.

The hUVSD model has increased discriminability estimates compared to the hierarchical EVSD model by Lee (2008) and Lee and Wagenmakers (2013) with $\hat{\mu }_d$=1.92 for inductive reasoning and $\hat{\mu }_d$=3.28 for deductive reasoning (Table 9). Not surprisingly, compared to the hEVSD model, the criterion of the hUVSD is shifted approximately by half of the increase in discriminability.

A model comparison in terms of dic and waic suggests that the hUVSD model performed as good as the hEVSD model for inductive ($\Updelta$dic=2.6; $\Updelta$waic=-1.0) and deductive ($\Updelta$dic=0.5; $\Updelta$waic=-0.9) reasoning. Since predicted as well as observed hit and false-alarm rates were unreliable and resulted in extreme values, *z*-ROC graphs and predictive checks were omitted.

**Fig. 11:**
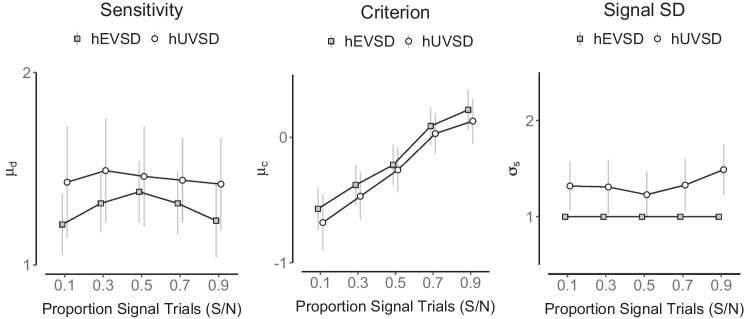
Sensitivity or discriminability (*left panel*), criterion (*middle panel*) and signal standard deviation (*right panel*) estimates of the hEVSD (*gray squares*) and hUVSD model (*white circles*) for data from Tanner et al. (1967). MCMC estimates are shown for five proportions of signal and noise trials ranging from S/N= 0.1 to 0.9. *Error bars* denote $\pm 1$ *SD* from the posterior mean (Table 10 and text for explanation)

In an attempt to benchmark the results of the hEVSD and hUVSD model, confidence ratings (1 to 7) for each response from the original data sets were incorporated (Heit and Rotello , personal communication). The response categories were collapsed into four categories with three thresholds to increase the number of observations for each category. The collapsed ratings were fit to hierarchical mixed-effect models with equal and unequal variance (hEVSDc, hUVSDc) implemented in STAN (R-packages brms and rstan). These models can estimate a correlation between discriminability and the median threshold. The results are shown in Table 9. The hUVSDc models gave similar parameter estimates compared to the hUVSD model except for an increased estimate of signal variance for inductive reasoning and a reduced estimate of discriminability for deductive reasoning. The correlations between discriminability and centre threshold ranged between -0.12 and 0.03 for inductive reasoning and 0.34 and 0.22 for deductive reasoning. Interestingly, the hierarchical model with unequal variance (hUVSDc) had a significantly lower waic than the corresponding equal-variance model (hEVSDc), suggesting that the unequal-variance model performed better. The estimates of signal SD are clearly larger than 1.0 for inductive and deductive reasoning. This suggests that hEVSD estimates of discriminability and criterion are biased and that both ‘strong’ and ‘valid’ arguments are better represented by a wider signal distribution for ‘weak’ as well as ‘invalid’ arguments.

Avoiding the difficult debate on how to best model inductive and deductive reasoning (e.g., Rotello et al., 2019), the results of the hUVSD model confirm previously established effects while parameter estimates of discriminability and criterion reflect increased signal variance.

### Auditory discrimination

In this classic data set from Tanner et al. (1967) a small sample of $n=12$ naive participants with normal hearing engaged in an auditory discrimination task. In each trial, a participant had to decide which of two auditory amplitudes had occurred. The two tones were 1,000 Hz sinusoids presented for 100 ms. The amplitude of the ‘loud’ tone had 70 dB whereas the ‘soft’ tone was adjusted individually for each participant so that they were 70% correct after practice trials (mean 67.5 dB, range 66.8–68.0 dB). In successive 5-day blocks of trials, each participant performed for 1 day in one of five conditions with different proportions of signal and noise stimuli, ranging from S/N= 0.1 to 0.9 in steps of 0.2.

The participants were given no information concerning the presentation schedules and no trial-to-trial feedback during the experiment (Tanner et al., 1967). The probability data were expressed as number of hit and false alarms adjusted to the number of signal and noise trials in the five S/N conditions. The data sets of each condition were analysed using the hEVSD model with four ($\sigma _s=\sigma _n=1$) and hUVSD model with five population parameters (Fig. 11 and Table 10).

A standard prediction for the five experimental conditions is that for increasing proportions of signal trials the criterion should shift from negative to positive values, describing step changes from more ‘lenient’ to more ‘stringent’ response thresholds. Discriminability, on the other hand, is expected to remain constant, showing approximately the same value across all five conditions.

Again, model performance in terms of dic was similar for both models. As predicted, the hEVSD and hUVSD model both show an increase of criterion estimates across the five conditions. In addition, the hUVSD model produces not only systematically lower criterion estimates and higher discriminability estimates but suggests more constant discriminability values across the five conditions. The hEVSD model, on the other hand, suggests a modulation of discriminability with a peak at S/N=0.5.

This was confirmed in secondary analyses using a linear mixed-effect model with subject-specific random intercepts (R-package lme4; Bates et al., 2015) on participants’ discriminability $\hat{d}_i$ and criterion $\hat{c}_i$ estimates for the two hierarchical signal detection models across the five conditions. For the criterion estimates, a statistically significant fixed effect (Satterthwaite’s method) of model (*t*(1)=-3.80, $p<0.0003$) and condition (*t*(1)=8.50, $p<0.0001$) was found. For the discriminability estimates, a statistically significant fixed effect for model (*t*(1)=5.15, $p<0.0001$) as well as an interaction between model and condition (*t*(1)=-2.04, $p=0.044$) emerged. The significant interaction suggests that the hUVSD and hEVSD model produce different discriminability estimates across conditions. The hUVSD model suggests approximately constant discriminability whereas the hEVSD model suggests an inverse ‘U shape’ of discriminability estimates with a peak at S/N=0.5.

The effects are illustrated in the left panel of Fig. 11 where posterior discriminability estimates for the hEVSD model peak at S/N=0.5 and are lower compared to the hUVSD model. The hUVSD model, on the other hand, has increased but relatively constant discriminability estimates. These results demonstrate that even for a small sample of $n=12$ participants the hUVSD model can provide more detailed information than the hEVSD model or non-hierarchical models (Table 10). If signal variance is allowed to vary across conditions (Fig. 11) then discriminability estimates are no longer significantly affected by the proportion of signal trials.

## Discussion

The hUVSD model assumes that individual sensitivity or discriminability $d_i$ and response criterion $c_i$ are drawn from independent normal distributions and that signal variance is approximately constant across participants. The resulting hierarchical Bayesian model takes advantage of information in the bivariate sampling distribution of (*z*-transformed) hit and false-alarm rates to estimate signal variance $\sigma ^2_s$.

Comparing population estimates between the hUVSD and hEVSD model in three applications confirms that a signal variance larger than 1.0 can lead to an increase of discriminability $\mu _d$ that is accompanied by a more lenient criterion $\mu _c$. Typically, the criterion is shifted to the left by about half the amount of the increase in discriminability. Performance measures $\texttt {dic}$ and $\texttt {waic}$ suggest equivalent performance because both models fit the observed hit and false-alarm rates equally well. However, taking into account distributional characteristics of the sampling distribution, in particular the ratio of variances between (*z*-transformed) hit and false-alarm rates, favour the hUVSD model.

For a sufficiently large sample of randomly selected participants with approximately constant signal variance the hUVSD model can estimate signal variance from the variability of hit and false-alarm rates. However, the population estimates are only an approximation and may not be as accurate as estimates in a hierarchical model that is based on estimates at the individual level (e.g., Pratte et al. 2010. Nevertheless, if signal and noise variances are not directly observable and unequal then the hUVSD model provides an alternative to the ubiquitous equal-variance model because potential bias in discriminability and criterion estimates is reduced.

The hUVSD model bridges the gap between hierarchical equal-variance models for yes-no responses (Lee & Wagenmakers, 2013) and hierarchical unequal-variance models based on confidence ratings with observable signal variance at the individual level (Pratte et al., 2010; Selker et al., 2019). It allows researchers to compare hEVSD and hUVSD parameters based on binary response data in a single condition. Performance can be evaluated for yes-no responses, dichotomised confidence ratings, and related measures in more complex paradigms if other relevant factors and mixed effects are considered. For certain tasks and more complex paradigms, it may be necessary to modify and extend the hUVSD model so that it can address specific hypotheses. Nevertheless, the present hierarchical unequal-variance model has a broad appeal and provides a good starting point for further developments.

Conditional and marginal posterior distributions have been derived in closed form for hierarchical models with unknown Gaussian mean and variance (e.g., Bernardo and Smith, 1994; Gelman et al. 2013), but the hUVSD model is more complex and has no known analytical solution (Bayesian or maximum likelihood) because individual discriminability and criterion values cannot be observed directly and rely on binomial parameters estimated from a sample of observed hit and false-alarm rates.

As stated by Wolpert and Macready (1997) there is no “free lunch” in optimisation and this also applies to fitting signal detection models to empirical data. The hUVSD model is no exception because it makes a number of critical assumptions: (1) Hit and false-alarm rates are modelled by binomial distributions relying on a sufficient number of independent trials. (2) Signal and noise variance may be unequal but should be approximately the same across participants. (3) Sensitivity or discriminability and criterion values vary independently and normally in a single condition and for a sufficiently large sample of randomly selected participants.

Each of these assumptions is plausible but have the following limitations: Ad (1): Any sequential dependency or auto-correlation between trials challenges the assumption of binomially distributed responses. Ad (2): It is well known (e.g., Egan, 1975) that the ratio of signal and noise standard deviations depends on the underlying distributions of the decision model. For example, log-normal, gamma, or double-exponential signal and noise distributions may have the same operational characteristic but produce different ratios of signal and noise standard deviations, which can make it difficult to distinguish between families of response functions (Rouder et al., 2010; Wixted & Mickes, 2010). It seems likely that not only signal variance but also noise variance varies across participants. However, variable signal and noise variance can be approximated by their ratio. Ad (3): The assumption of independent normal distributed discriminability and criterion values is plausible for a single condition but difficult to verify in more complex designs. Assuming independence between discriminability and criterion among randomly selected participants may be less contentious than assuming constant signal (and noise) variance across conditions in a within-subjects design.

The hUVSD model is more flexible than the hEVSD model, but requires that hit and false-alarm rates can be estimated from a sufficient number of participants to reliably estimate signal variance together with the other population parameters. It is usually easier to increase the sample size of participants than to gather additional data from the same participant in multiple conditions and/or sessions where contextual effects are likely to affect parameters.

Although the constraints of the hUVSD model are plausible, the signal variance parameter introduces dependencies between discriminability, criterion and signal variance estimates. This does not invalidate the model but can affect MCMC sampling and estimation. If parameter estimates of the hUVSD model are correlated then a Metropolis–Hamiltonian (STAN) rather than a Gibbs sampler (JAGS) may improve sampling and convergence. Therefore, the hUVSD and hEVSD model were implemented in STAN and applied to the memory and reasoning data using package rstan in R. Running the hUVSD and hEVSD model for JAGS and STAN gave almost identical parameter estimates for the dichotomised memory data (Table 8) and similar estimates for the reasoning data (Table 9). Convergence diagnostics in terms of $\hat{R}$, efficiency, and auto-correlation of posterior estimates were more reliable for STAN compared to JAGS.

It is important to acknowledge that not only sampling of participants but also sampling of items and other random factors can contribute to variability in observed data (Baayen et al., 2008; Rouder and Lu, 2005; DeCarlo, 2011; Rabe et al., preprint). Similarly, introducing additional conditions, tasks, or response categories are likely to affect performance and therefore parameter estimation. A possible influence that is not elaborated here are sequential dependencies between trials that can affect responses (Lages, 2002; Lages & Treisman, 2010; Treisman & Lages, 2010; Lages & Jaworska, 2012). These stimulus, task and design-specific issues warrant careful inspection of the data before applying any signal detection model, including the hUVSD model. Nevertheless, the present simulations and applications suggest that the hUVSD model offers a robust alternative that can improve fits and may reveal novel and more detailed information than the ubiquitous equal-variance model.

## Conclusion

A hierarchical signal detection models with equal variance can be extended to a model with unequal variance by imposing plausible constraints on the distribution of sensitivity or discriminability and response criterion at the population level. The hierarchical signal detection model with unequal variance was investigated analytically, explored in simulations and applied to existing data sets in three different domains. Analysing dichotomised confidence ratings of word recognition, inductive and deductive reasoning, and binary data of auditory discrimination of loudness provided encouraging results.

This work serves as a proof-of-concept and the simulations suggest that the hUVSD model performs well as long as sample sizes and trial numbers are sufficiently large and individual sensitivity and criterion are independent. In the present applications, parameter estimates differed significantly between the hUVSD and hEVSD model. The empirical results indicate that the hUVSD model with unequal variance is more flexible and may produce less biased estimates than the hEVSD model with equal variance when plausible assumptions are met. It is concluded that for binary data the hUVSD model provides a promising alternative to the ubiquitous equal-variance models.

### Open practices statement

This article is distributed under the terms of the Creative Commons Attribution 4.0 International License (http://creativecommons.org/licenses/by/4.0), which permits unrestricted use, distribution, and reproduction in any medium, provided you give appropriate credit to the original author(s) and the source, provide a link to the Creative Commons license, and indicate if changes were made.

Data and code for analyses, simulations and figures are available at the OSF website https://osf.io/dsygh.

## Figures and Tables

**Fig. 12:**
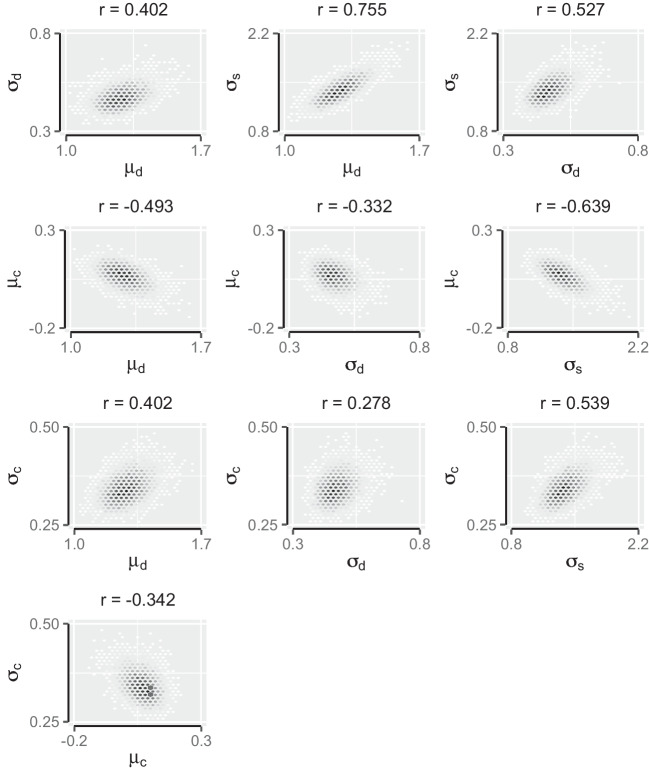
MCMC parameter estimates (JAGS) of the hUVSD model for recognition data by Pratte et al. (2010). Bivariate hexagon plots for all pairs of parameters at the population level. *Light to dark hexagons* indicate increasing frequencies. Pearson correlations between posterior samples are shown on top of each panel

**Fig. 13:**
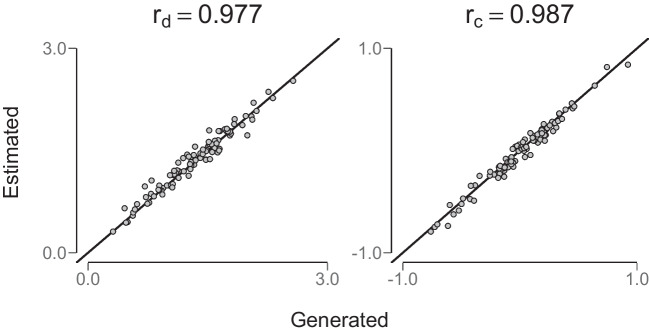
Parameter recovery for discriminability $d_i$ (*left panel*) and criterion $c_i$ (*right panel*) from synthetic data generated with population parameters, *k*=240 trials and *n*=97 participants as in Pratte et al. (2010)

**Fig. 14:**
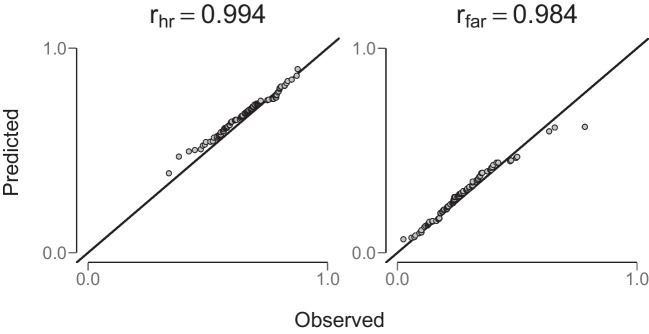
Posterior predictive check for hit and false-alarm rates using observed data and hUVSD population estimates from Pratte et al. (2010). Predicted and observed hit rates (*left panel*) as well as predicted and observed false-alarm rates (*right panel*) were independently rank-ordered for simulated and real participants

**Fig. 15:**
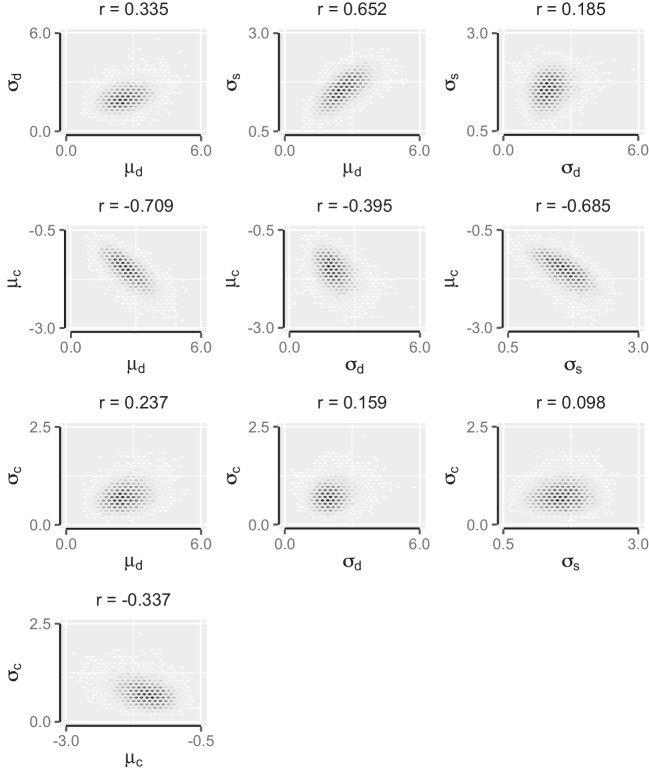
MCMC parameter estimates (JAGS) of the hUVSD model for inductive reasoning data by Heit and Rotello (2005). Bivariate hex plots for parameters pairs at the population level. *Light to dark hexagons * indicate increasing frequencies. Pearson correlations between posterior samples are shown on top of each panel

**Fig. 16:**
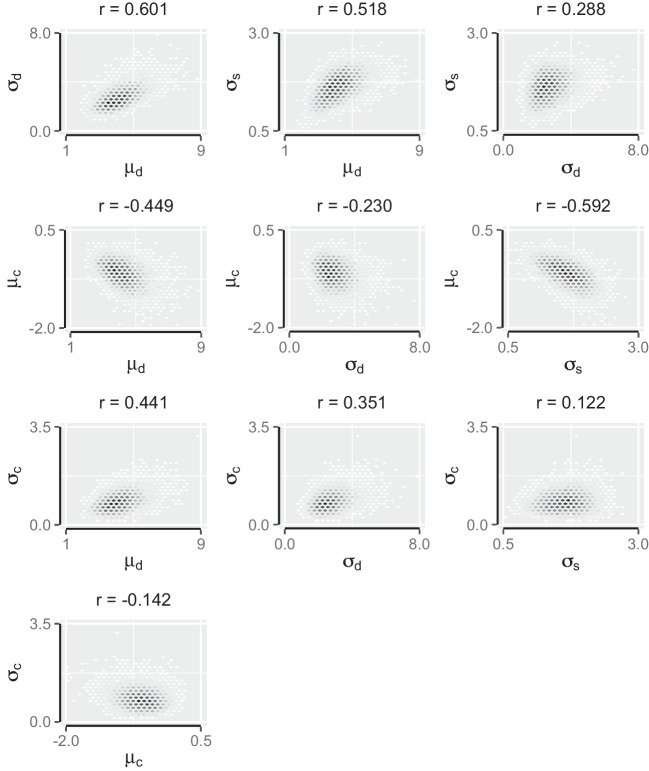
MCMC parameter estimates (JAGS) of the hUVSD model for deductive reasoning data by Heit and Rotello (2005). Bivariate hex plots for all pairs of parameters at the population level. *Light to dark hexagons* indicate increasing frequencies. Pearson correlations between posterior samples are shown on top of each panel

## Appendix A: Analytical results

Proposition 1 establishes the correspondence between the five parameters of the sampling distribution and the five re-parameterised population parameters.

In Proposition 2 it is shown that independence between normal distributed discriminability and criterion is necessary and sufficient to estimate parameter $\sigma _s$ in the hUVSD model.

Let $z(\theta ^h) = (z(\theta ^h_1), \ldots , z(\theta ^h_n) )$ denote a random sample of transformed hit rates and $z(\theta ^f) = (z(\theta ^f_1), \ldots , z(\theta ^f_n) )$ denote the corresponding random sample of transformed false-alarm rates. If the vectors $d=(d_1, \dots , d_n)$ and $c=(c_1,\dots , c_n)$ contain independent samples from normal distributions then for $n \rightarrow \infty$ the variances are additive and covariance $\text {cov}[d,c] = \sigma _{d,c}=0$.

### Proposition 1

Assume a hUVSD model with Gaussian signal and noise distributions, then for $n \rightarrow \infty$

$$\begin{aligned} \text {E}\,[z(\theta ^h)]= & {} (\mu _d/2 - \mu _c)/ \sigma _s \\ \text {var}[z(\theta ^h)]= & {} (\sigma ^2_c + \sigma ^2_d/4)/\sigma ^2_s \\ \text {E}\,[z(\theta ^f)]= & {} - \mu _d/2 - \mu _c \\ \text {var}[z(\theta ^f)]= & {} \sigma ^2_c + \sigma ^2_d/4 \\ \text {cov}[z(\theta ^h),z(\theta ^f)]= & {} (\sigma ^2_c - \sigma ^2_d/4)/ \sigma _s \\ \end{aligned}$$

### Proof

The expected values of the transformed hit rate and false-alarm rate follow from Eq. 4, assuming independence between *d* and *c*.

$$\begin{aligned} \text {var}[z(\theta ^h)]= & {} \text {var}[(0.5\,d - c)/ \sigma _s] \\= & {} \left( \text {var}[0.5\,d] + \text {var}[c] - 2 \, \text {cov}[0.5\,d, c] \right) 1/\sigma ^2_s \\= & {} (\sigma ^2_d/4 + \sigma ^2_c) /\sigma ^2_s \\ \text {var}[z(\theta ^f)]= & {} \text {var}[(-0.5\,d - c)] \\= & {} \text {var}[-0.5\,d] + \text {var}[c] - 2 \, \text {cov}[-0.5\,d, c] \\= & {} \sigma ^2_d/4 + \sigma ^2_c \\ \end{aligned}$$

From the bilinear property of covariances and (4) follows that

$$\begin{aligned} \text {cov}[z(\theta ^h),z(\theta ^f)]= & {} \text {cov}[ (0.5\,d - c)/ \sigma _s, -0.5\,d - c ] \\= & {} -0.25/ \, \sigma _s \, \text {cov}[d,d] -0.5 / \sigma _s \, \text {cov}[d,c] + \ldots \\{} & {} \ldots + 0.5 / \sigma _s \, \text {cov}[c,d] + 1/\sigma _s \text {cov}[c,c] \\= & {} -1/(4 \sigma _s) \, \text {var}[d] + 1/ \sigma _s \, \text {var}[c] \\= & {} (\sigma ^2_c - \sigma ^2_d/4)/ \sigma _s . \end{aligned}$$

$$\square $$

### Proposition 2

Assume a hUVSD model with Gaussian signal and noise distributions then for $n \rightarrow \infty$ the ratio of sample variances $\text {var}[z(\theta ^f)]$/$\text {var}[z(\theta ^h)]$ equals signal variance $\sigma ^2_s$ if and only if $d_i \sim \mathcal {N}(\mu _d, \sigma _d)$ and $c_i \sim \mathcal {N}(\mu _c, \sigma _c)$ are sampled from independent normal distributions.

### Proof

This follows from Proposition 1. Since $\sigma _s \, z(\theta _h) = +0.5\, d - c$ and $\sigma _n \, z(\theta _f) = -0.5\, d - c$, with pairwise independent $d_i$ and $c_i$ the two variances $\text {var}[+0.5\, d - c]$ and $\text {var}[-0.5 \, d - c]$ are equal. It follows that

$$\begin{aligned} \text {var}[\sigma _s \, z(\theta ^h)]= & {} \text {var}[\sigma _n \, z(\theta ^f)] \\ \sigma ^2_s \, \text {var}[z(\theta ^h)]= & {} \sigma ^2_n \, \text {var}[z(\theta ^f)] \\ \sigma ^2_s/\sigma ^2_n= & {} \text {var}[z(\theta ^f)]/\text {var}[z(\theta ^h)] \end{aligned}$$

If discriminability and criterion values are sampled from independent normal distributions and $\sigma _n=1$ then $\sigma _s$ can be approximated by $\text {sd}[z(\theta ^f)]/\text {sd} [z(\theta ^h)]$. Conversely, using the bilinear property of the covariance, it is straightforward to show that $\sigma ^2_s = \text {var}[z(\theta ^f)]/\text {var}[z(\theta ^h)]$ implies $\text {cov}[d,c] = 0$. $\square$

### Marginal posterior distributions

A family of *t*-distributions, $t_{\nu }(\mu , \sigma ^2)$, is characterised by three parameters: location $\mu$, scale $\sigma ^2$, and degree of freedom $\nu$ which determines the shape of the distribution. The densities of *t*-distributions are symmetric, and $\nu$ must fall in the range $(0,\infty )$. For $\nu =1$, the *t*-distribution is equivalent to the Cauchy distribution with infinite mean and variance. As $\nu \rightarrow \infty$ the *t*-distribution approximates the normal distribution. Generalised Student’s *t*-distributions have heavier tails than the standard normal distribution and feature frequently in hierarchical Bayesian models because they can accommodate outliers or extreme parameter estimates (e.g., Gelman et al., 2013) and because of their relation to the normal distribution: When the precision (variance) of a normally distributed random variable is unknown and a conjugate prior is placed on the precision (variance) that follows an (inverse-)gamma distribution then the marginal posterior has a Student’s *t*-distribution (e.g., Johnson and Kotz, 1972; Bishop, 2006; Gelman, 2006; Gelman et al., 2013). Similarly, the marginal posterior of the precision (variance) follows a (inverse-)gamma distribution.

In the context of the hUVSD model, compounding a normal distribution with a gamma-distributed variance, the marginal posterior distributions for population mean $\mu _d$ and $\mu _c$ are also *t*-distributed whereas for variance $\sigma ^2_d$ and $\sigma ^2_c$ marginal posterior distributions follow gamma distributions. However, the (marginal) posterior distributions are difficult to derive analytically. This is the case because population estimates of $\mu _d$ and $\sigma _d$ as well as $\mu _c$ and $\sigma _c$ depend on samples of individual estimates $d_i$ and $c_i$. These estimates are not directly observable but are linear functions of the transformed estimate $z(\theta ^h_i)$, scaled by $\sigma _s$, and false-alarm rate estimate $z(\theta ^f_i)$, multiplied by $\sigma _n$. Estimates are therefore conditional on the (*z*-transformed) binomial hit and false-alarm rates, their variances and covariance for a given number of trials.

## Appendix B: Simulation results

**Table 1:** Posterior means and 95% credible intervals in brackets estimated from simulated data with sample size *n*=256, signal and noise trial number *k*=256

	Parameter recovery of hUVSD model for seven different criterion values $\mu _c$				
$\mu _c$	$\hat{\mu }_d$ (1.5)	$\hat{\mu }_c$ (−0.75 to +0.75)	$\hat{\sigma }_d$ (0.5)	$\hat{\sigma }_c$ (0.25)	$\hat{\sigma }_s$ (1.25)
−0.75	1.51 [1.31,1.77]	$-0.75 [-0.87,-0.65]$	0.53 [0.47,0.59]	0.27 [0.24,0.30]	1.26 [1.10,1.48]
−0.50	1.55 [1.36,1.75]	$-0.56 [-0.66,-0.47]$	0.54 [0.48,0.61]	0.25 [0.23,0.28]	1.33 [1.15,1.51]
−0.25	1.56 [1.41,1.72]	$-0.31 [-0.39,-0.24]$	0.48 [0.43,0.55]	0.25 [0.22,0.28]	1.38 [1.21,1.58]
$\pm 0.0$	1.53 [1.41,1.67]	$-0.05 [-0.12,0.01]$	0.53 [0.47,0.59]	0.26 [0.23,0.29]	1.32 [1.17,1.51]
+0.25	1.63 [1.53,1.74]	0.23 [0.17,0.28]	0.51 [0.46,0.58]	0.30 [0.26,0.33]	1.48 [1.29,1.69]
+0.50	1.47 [1.40,1.55]	0.48 [0.44,0.52]	0.52 [0.46,0.58]	0.27 [0.24,0.31]	1.31 [1.15,1.50]
+0.75	1.50 [1.44,1.57]	0.75 [0.71,0.78]	0.50 [0.45,0.57]	0.27 [0.24,0.31]	1.31 [1.15,1.50]

Hit and false-alarm rates were generated from population parameter values as shown in parentheses in the top row with $\mu _c$ ranging from -0.75 to +0.75 in steps of 0.25. The 95% CrI[,] is underlined if the true value is outside the interval

**Table 2:** Posterior means and 95% credible intervals in brackets estimated from simulated data with sample size *n*=500, trial number *k*=200 (100 signal and 100 noise trials)

	Parameter recovery of hUVSD model for five different discriminability values $\mu _d$				
$\mu _d$	$\hat{\mu }_d$ (0.5 to 2.5)	$\hat{\mu }_c$ (-0.25)	$\hat{\sigma }_d$ (1.25)	$\hat{\sigma }_c$ (0.75)	$\hat{\sigma }_s$ (1.5)
0.5	$0.62 [0.50-0.74]$	$-0.24 [-0.31,-0.17]$	1.25 [1.15,1.35]	0.75 [0.70,0.81]	1.48 [1.36,1.63]
1.0	1.09 [0.97,1.23]	$-0.24 [-0.32,-0.17]$	1.26 [1.17,1.37]	0.75 [0.70,0.81]	1.50 [1.38,1.63]
1.5	1.60 [1.46,1.75]	$-0.24 [-0.32,-0.16]$	1.25 [1.16,1.35]	0.75 [0.69,0.81]	1.50 [1.37,1.63]
2.0	2.10 [1.95,2.26]	$-0.25 [-0.33,-0.17]$	1.23 [1.14,1.33]	0.74 [0.69,0.80]	1.48 [1.37,1.61]
2.5	$2.58 [2.41-2.76]$	$-0.24 [-0.33,-0.15]$	1.24 [1.14,1.34]	0.74 [0.69,0.80]	1.48 [1.35,1.61]

Hit and false-alarm rates were generated from population parameter values as shown in parentheses in the top row with $\mu _d$ ranging from 0.5 to 2.5 in steps of 0.5

**Table 3:** Posterior means and 95% credible intervals in brackets estimated from simulated data with sample size *n*=500, trial number *k*=200 (100 signal and 100 noise trials)

	Parameter recovery of hUVSD model for five different signal *SD* values $\sigma _s$				
$\sigma _s$	$\hat{\mu }_d$ (2.0)	$\hat{\mu }_c$ (-0.5)	$\hat{\sigma }_d$ (0.75)	$\hat{\sigma }_c$ (0.5)	$\hat{\sigma }_s$ (0.5 to 2.5)
0.5	$2.18 [\underline{2.02,-2.37}]$	$-0.60 [\underline{-0.70,-0.51}]$	0.76 [0.70,0.83]	0.52 [0.48,0.56]	0.57 [0.50,0.64]
1.0	2.08 [1.92,2.26]	$-0.54 [-0.63,-0.45]$	0.74 [0.68,0.80]	0.52 [0.48,0.56]	1.05 [0.95,1.15]
1.5	2.09 [1.95,2.26]	$-0.56 [-0.65,-0.49]$	0.74 [0.68,0.80]	0.52 [0.48,0.56]	1.59 [1.47,1.74]
2.0	2.04 [1.90,2.20]	$-0.53 [-0.61,-0.45]$	0.74 [0.68,0.80]	0.51 [0.47,0.55]	2.05 [1.88,2.23]
2.5	1.95 [1.81,2.09]	$-0.47 [-0.55,-0.40]$	0.71 [0.66,0.77]	0.50 [0.47,0.54]	2.38 [2.19,2.59]

Hit and false-alarm rates were generated from population parameter values as shown in parentheses in the top row with $\sigma _s$ ranging from 0.5 to 2.5 in steps of 0.5. The 95% CrI[,] is underlined if the true value is outside the interval

**Table 4:** Estimated posterior means and 95% credible intervals in brackets for simulated data with decreasing sample size *n* and trial number *k* generated from population parameter values as shown in the top row with variable $\sigma _{s,i} \sim \mathcal {N}(\sigma _s, 2 \sigma _s^4/n)$ and a correlation of +0.5 between $d_i$ and $\sigma _{s,i}$

	Parameter recovery of hUVSD Model for participants *n* and trials *k*, ($d_i$ correlated with $\sigma _{s,i}$)					
*n*	*k*	$\hat{\mu }_d$ (2.0)	$\hat{\mu }_c$ (-0.5)	$\hat{\sigma }_d$ (0.6)	$\hat{\sigma }_c$ (0.4)	$\hat{\sigma }_s$ (1.5)
512	512	1.98 [1.85,2.10]	$-0.46 [-0.53,-0.39]$	0.55 [0.51,0.59]	0.41 [0.38,0.44]	1.48 [1.37,1.59]
256	256	1.99 [1.85,2.16]	$-0.44 [-0.53,-0.36]$	0.54 [0.49,0.61]	0.42 [0.38,0.47]	1.46 [1.34,1.63]
128	128	1.93 [1.68,2.23]	$-0.44 [-0.60,-0.30]$	0.53 [0.44,0.63]	0.43 [0.37,0.51]	1.37 [1.14,1.65]
64	64	2.04 [1.67,2.45]	$-0.45 [-0.68,-0.25]$	0.61 [0.48,0.79]	0.52 [0.42,0.64]	1.49 [1.16,1.88]
32	32	2.05 [1.54,2.61]	$-0.52 [-0.85,-0.23]$	0.57 [0.39,0.76]	0.55 [0.41,0.76]	1.58 [1.10,2.10]
16	16	2.13 [1.32,3.09]	$-0.57 [-1.06,-0.16]$	0.85 [0.49,1.53]	0.48 [0.31,0.78]	1.46 [0.84,2.09]

The 95% CrI[,] it is underlined if the true value is outside the interval

**Table 5:** Estimated posterior means and 95% credible intervals in brackets for simulated data with decreasing sample size *n* and trial number *k* generated from population parameter values as shown in the top row with variable $\sigma _{s,i} \sim \mathcal {N}(\sigma _s,2 \sigma _s^4/n)$ and a correlation of +0.5 between $d_i$ and $\sigma _{s,i}$

	Parameter estimates of hEVSD Model for participants *n* and trials *k*, ($d_i$ correlated with $\sigma _{s,i}$)					
*n*	*k*	$\hat{\mu }_d$ (2.0)	$\hat{\mu }_c$ (-0.5)	$\hat{\sigma }_d$ (0.6)	$\hat{\sigma }_c$ (0.4)	$\sigma _s$ (1.5)
512	512	1.51 [1.46,1.55]	$-0.27 [\underline{-0.26,-0.20}]$	0.48 [0.45,0.51]	0.35 [ 0.32,0.37]	(1.0)
256	256	1.54 [1.48,1.60]	$-0.21 [\underline{-0.26,-0.17}]$	0.47 [0.43,0.52]	0.36 [0.33,0.39]	(1.0)
128	128	1.54 [1.45,1.63]	$-0.19 [\underline{-0.25,-0.11}]$	0.46 [0.40,0.53]	0.39 [0.34,0.45]	(1.0)
64	64	1.56 [1.43,1.69]	$-0.24 [\underline{-0.35,-0.14}]$	0.47 [0.38,0.59]	0.40 [0.33,0.49]	(1.0)
32	32	1.60 [1.37,1.84]	$-0.30 [\underline{-0.47,-0.14}]$	0.53 [0.37,0.75]	0.41 [0.31,0.56]	(1.0)
16	16	1.66 [1.18,2.20]	$-0.25 [-0.62, -0.08]$	0.78 [0.46,1.30]	0.45 [0.30,0.71]	(1.0)

The 95% CrI[,] is underlined if the true value is outside the interval

**Table 6:** Estimated posterior means and 95% credible intervals in brackets for simulated data with decreasing sample size *n* and trial number *k* generated from population parameter values as shown in the top row with variable $\sigma _{s,i} \sim \mathcal {N}(\sigma _s, 2 \sigma _s^4/n)$ and correlation of +0.5 between $c_i$ and $\sigma _{s,i}$

	Parameter recovery of hUVSD Model for participants *n* and trials *k*, ($c_i$ correlated with $\sigma _{s,i}$)					
*n*	*k*	$\hat{\mu }_d$ (2.0)	$\hat{\mu }_c$ (-0.5)	$\hat{\sigma }_d$ (0.6)	$\hat{\sigma }_c$ (0.4)	$\hat{\sigma }_s$ (1.5)
512	512	1.96 [1.84,2.08]	$-0.46 [-0.53,-0.40]$	0.59 [0.55,0.64]	0.39 [0.36,0.42]	1.41 [1.32,1.52]
256	256	2.00 [1.78,2.23]	$-0.46 [-0.57,-0.35]$	0.61 [0.55,0.69]	0.40 [0.36,0.44]	1.43 [1.24,1.64]
128	128	1.86 [1.61,2.14]	$$-0.39 [-0.54,-0.25] $$	0.60 [0.51,0.71]	0.38 [0.32,0.44]	1.27 [1.05,1.52]
64	64	1.89 [1.52,2.32]	$-0.43 [-0.65,-0.25]$	0.66 [0.52,0.85]	0.39 [0.31,0.49]	1.27 [0.96,1.63]
32	32	1.73 [1.23,2.37]	$$ -0.44 [-0.75,-0.17]$$	0.62 [0.42,0.90]	0.37 [0.27,0.52]	1.18 [0.76,1.71]
16	16	1.99 [1.34,2.74]	$-0.54 [-0.98,-0.15]$	0.58 [0.34,1.04]	0.52 [0.34,0.85]	1.64 [1.07,2.19]

All true values are inside the 95% CrI [,]

**Table 7:** Estimated posterior means and 95% credible intervals in brackets for simulated data with decreasing sample size *n* and trial number *k* generated from population parameter values as shown in the top row with a correlation of +0.5 between $d_i$ and $c_i$

	Parameter recovery of hUVSD Model for participants *n* and trials *k* ($d_i$ correlated with $c_i$)					
*n*	*k*	$\hat{\mu }_d$ (2.0)	$\hat{\mu }_c$ (-0.5)	$\hat{\sigma }_d$ (0.6)	$\hat{\sigma }_c$ (0.4)	$\sigma _s$ (1.5)
512	512	2.93 [2.70,3.13]	$-0.96 [\underline{-1.07,-0.85}]$	0.70 [0.65,0.75]	0.48 [0.45,0.52]	2.43 [2.22,2.61]
256	256	2.84 [2.60,3.13]	$-0.92 [\underline{-1.07,-0.80}]$	0.68 [0.61,0.76]	0.49 [0.44,0.54]	2.34 [2.11,2.61]
128	128	2.68 [2.38,3.03]	${-0.84} [\underline{-1.02,-0.68}]$	0.69 [0.59,0.81]	0.49 [0.42,0.56]	2.18 [1.91,2.50]
64	64	2.42 [2.01,2.89]	${-0.72} [-0.97,-0.50]$	0.61 [0.47,0.79]	0.46 [0.38,0.58]	1.89 [1.52,2.32]
32	32	2.21 [1.69,2.74]	${-0.64} [-0.92,-0.36]$	0.57 [0.38,0.85]	0.42 [0.31,0.59]	1.86 [1.37,2.34]
16	16	1.97 [1.32,2.71]	$-0.48 [-0.91, -0.07]$	0.64 [0.37,1.13]	0.55 [0.36,0.88]	1.56 [1.00,2.13]

The 95% CrI[,] is underlined if the true value is outside the interval

## Appendix C: Application results

**Table 8:** Population parameter estimates of hierarchical models for word recognition task (Pratte et al., 2010). Models from Pratte et al. (2010) and Selker et al. (2019) are based on confidence ratings and have different parameters (n/a)

	Parameter Estimates for Word Recognition Task							
Model	sampler	dic/waic	$A_z$	$\hat{\mu }_d$	$\hat{\mu }_c$	$\hat{\sigma }_d$	$\hat{\sigma }_c$	$\hat{\sigma }_s$
Pratte et al.	n/a	n/a	0.793	1.38	n/a	n/a	n/a	1.36
Selker et al.	JAGS	n/a	0.778	1.41	n/a	n/a	n/a	1.54
hEVSD	JAGS	1470	0.785	1.12	0.15	0.42	0.31	(1.0)
hUVSD	JAGS	1469	0.771	1.31	0.05	0.49	0.36	1.45
hEVSD	STAN	1422	0.788	1.13	0.15	0.42	0.31	(1.0)
hUVSD	STAN	1422	0.772	1.32	0.05	0.49	0.36	1.46

Reported parameter estimates are the result of Gibbs (JAGS) and Metropolis–Hamiltonian (STAN) MCMC sampling, reporting dic and waic, respectively

**Table 9:** Population parameter estimates for inductive and deductive reasoning task by Heit and Rotello (2005)

	Parameter Estimates for Reasoning Tasks							
Model	sampler	waic	$A_z$	$\hat{\mu }_d$	$\hat{\mu }_c$	$\hat{\sigma }_d$	$\hat{\sigma }_c$	$\hat{\sigma }_s$
Inductive reasoning								
hEVSDc	STAN	541.8	0.927	2.06	$-1.14$	1.54	0.55	(1.0)
hUVSDc	STAN	520.3	0.842	2.58	$-1.40$	1.39	0.47	2.37
hEVSD	STAN	154.2	0.916	1.95	$-1.19$	1.88	0.76	(1.0)
hUVSD	STAN	155.2	0.924	2.93	$-1.70$	2.06	0.82	1.78
Deductive reasoning								
Model	sampler	waic	$A_z$	$\hat{\mu }_d$	$\hat{\mu }_c$	$\hat{\sigma }_d$	$\hat{\sigma }_c$	$\hat{\sigma }_s$
hEVSDc	STAN	485.7	0.952	2.36	$-0.34$	1.31	0.38	(1.0)
hUVSDc	STAN	408.9	0.944	3.13	$-0.53$	1.94	0.47	1.70
hEVSD	STAN	134.9	0.991	3.37	$-0.31$	2.30	0.80	(1.0)
hUVSD	STAN	135.8	0.984	4.40	$-0.71$	2.74	0.88	1.80

Parameter estimates and waic are the result of Metropolis–Hamiltonian (STAN) MCMC sampling

**Table 10:** Population parameter estimates of hEVSD and hUVSD model for auditory discrimination data from Tanner et al. (1967)

	Parameter Estimates for Auditory Discrimination Task							
Model	S/N	dic	$A_z$	$\hat{\mu }_d$	$\hat{\mu }_c$	$\hat{\sigma }_d$	$\hat{\sigma }_c$	$\hat{\sigma }_s$
hEVSD	0.1	180.9	0.804	1.21	-0.57	0.50	0.59	(1.0)
hUVSD		181.7	0.821	1.43	-0.68	0.48	0.57	1.19
hEVSD	0.3	194.9	0.825	1.32	-0.38	0.47	0.52	(1.0)
hUVSD		195.2	0.833	1.49	-0.47	0.44	0.50	1.17
hEVSD	0.5	197.5	0.835	1.38	-0.22	0.54	0.55	(1.0)
hUVSD		197.9	0.838	1.46	-0.26	0.52	0.51	1.09
hEVSD	0.7	197.0	0.825	1.32	0.09	0.54	0.51	(1.0)
hUVSD		197.1	0.821	1.44	0.03	0.54	0.49	1.21
hEVSD	0.9	186.1	0.808	1.23	0.22	0.64	0.54	(1.0)
hUVSD		186.2	0.789	1.42	0.13	0.69	0.57	1.46

In five conditions the proportion of signal trials was increased from S/N=0.1 to 0.9 in steps of 0.2. Parameter estimates are the result of Gibbs (JAGS) MCMC sampling

## Footnotes

1 Please note that in some publications ’individual’ or ‘participant’ level is labelled ‘group’ level, whereas in other publications ‘group’ level refers to the ‘population’ level.
